# Low-Temperature Superplasticity and Deformation Mechanism of Ti-6Al-4V Alloy

**DOI:** 10.3390/ma11071212

**Published:** 2018-07-13

**Authors:** Ge Zhou, Lijia Chen, Lirong Liu, Haijian Liu, Heli Peng, Yiping Zhong

**Affiliations:** 1 School of Materials Science and Engineering, Shenyang University of Technology, Shenyang 110870, China; zhouge19850131@163.com (G.Z.); liulirong@sut.edu.cn (L.L.); 2 Research and development center, Shanghai Spaceflight Precision Machinery Institute, Shanghai 201600, China; hangtian402@163.com (H.L.); phl12616040811@126.com (H.P.); lizhongquan401@163.com (Y.Z.)

**Keywords:** Ti-6Al-4V alloy, deformation activation energy, strain rate sensitivity exponent, low-temperature superplasticity, deformation mechanism map

## Abstract

The low-temperature superplastic tensile behavior and the deformation mechanisms of Ti-6Al-4V alloy are investigated in this paper. Through the experiments carried out, elongation to failure (*δ*) is calculated and a set of values are derived that subsequently includes the strain rate sensitivity exponent (*m*), deformation activation energy (*Q*) at low-temperature superplastic deformation, and the variation of *δ*, *m* and *Q* at different strain rates and temperatures. Microstructures are observed before and after superplastic deformation. The deformation mechanism maps incorporating the density of dislocations inside grains at temperatures of 973 and 1123 K are drawn respectively. By applying the elevated temperature deformation mechanism maps based on Burgers vector compensated grain size and modulus compensated stress, the dislocation quantities and low-temperature superplastic deformation mechanisms of Ti-6Al-4V alloy at different temperatures within appropriate processing regime are elucidated.

## 1. Introduction

The two-phase α-β Ti-6Al-4V alloy has been widely used for aerospace applications because of an attractive combination of properties such as high specific strength, relatively excellent fracture toughness, and strong heat and corrosion resistance [1,2,3]. However, the alloy is rather difficult to form into a complex shape at room temperature because of its poor formability, and thus the characterization of the deformation behavior during superplastic forming is essential for optimizing hot forged processes of titanium alloys [4]. Moreover, the oxidation resistance of conventional titanium alloys decreases sharply during high-temperature superplasticity [5]. Therefore, the low-temperature superplasticity makes it possible to improve the oxidation resistance of titanium alloys, and to reduce cost in superplastic forming industries. By far, there is still little research that concerns the low-temperature superplasticity and deformation mechanism of the Ti-6Al-4V alloy.

The superplasticity deformation mechanisms under given conditions can be identified according to some workability theories, especially a deformation mechanism map based on dislocation kinetics and metallographic theory. The deformation mechanism map has been successfully applied to stainless steel, aluminum and aluminum composites, magnesium alloy, nickel and its alloy, and titanium etc. [6,7,8,9,10,11,12,13,14]. For example, Ashby [6] introduced a deformation mechanism map showing the area of dominant flow mechanisms in the plot of normalized stress vs. temperature for a given grain size to predict creep deformation of tungsten filament in bulb. Mohamed et al. [7] and Langdon et al. [5] constructed a deformation mechanism map as a function of strain size, stress, and temperature applicable to aluminum. Ruano et al. [8] also proposed a grain size-dependent deformation mechanism map for predicting the superplastic grain boundary mechanism of a Fe-25Cr-20Ni austenite stainless steel.

Recently, studies and reports on deformation mechanism diagrams have shown that aluminum base composite, nickel base superalloy GTD-111, industrial titanium alloy CP-Ti, and AZ61 magnesium alloy can also be mapped [15,16,17]. In practice, these maps were developed and applied on the basis of the previous studies [18,19,20,21,22]. However, little work has discussed the deformation mechanism map incorporating a dislocation quantity inside the grains.

A deformation mechanism map proposes a new method in discovering quantitatively the essence of a rate controlling process. The dislocation creep mechanism map of a solid solution alloy was ascertained by two regimes; i.e., dislocation viscous glide mechanism regime and dislocation climb mechanism regime. The dislocation breakaway solute atmosphere mechanism regime in the Langdon model is not included in the above deformation mechanism map. Therefore, it is necessary to construct a new type of deformation mechanism map for solid solution alloys.

In this study, therefore, the low-temperature superplasticity and deformation behavior of Ti-6Al-4V alloy was investigated on the basis of the flow curves measured during tensile tests at different tensile temperatures with different strain rates. A new deformation mechanism map containing the dislocation quantity was constructed for the two-phase titanium alloy to predict its low-temperature deformation mechanism, and to analyze quantitatively the dislocation quantity during deformation. The results obtained in the present study can provide a theoretical basis for the application of low-temperature superplasticity of Ti-6Al-4V alloy.

## 2. Experimental

**Figure 1:**
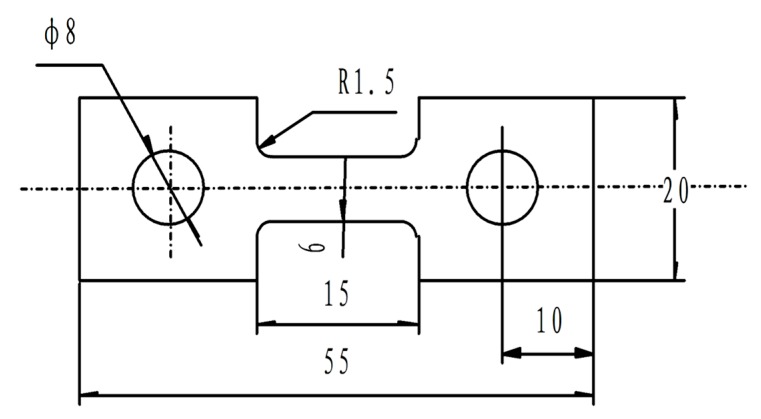
Schematic diagram of superplastic tension specimen (Unit: mm).

The material used for the superplastic tensile tests was as a cold-rolled Ti-6Al-4V alloy sheet with a thickness of 2 mm. The gauge part of the superplastic tensile specimens was 6 mm in width, 15 mm in length, and 2 mm in thickness, as shown in Figure 1. Superplastic tensile tests were performed in an argon atmosphere under uniform heating conditions using a computer-controlled servo-hydraulic testing machine (MTS, Shanghai, China). After heated to the desired temperature and held for 10 min, the specimen was deformed at a constant crosshead speed, followed by water-quenching to room temperature. The deformation temperature ranges from 973 to 1123 K at 50 K intervals and the strain rates are between 3 × 10^−4^ and 5 × 10^−3^ s^−1^.

The specimens for the metallographic examination were mechanically polished and etched in a mixed solution of 20 mL HF, 40 mL HNO_3_, and 100 mL H_2_O, and then were observed using an optical microscope (OM, LEICA Q550IW, LEICA Microsystems, Wetzlar, Germany). The mean grain size and volume fraction of the β phase were examined using an image-analyzer (OLYMPUS M3, Olympus Optical Co., Ltd, Tokyo, Japan).

## 3. Results and Discussion

### 3.1. Microstructure Prior to Deformation

**Figure 2:**
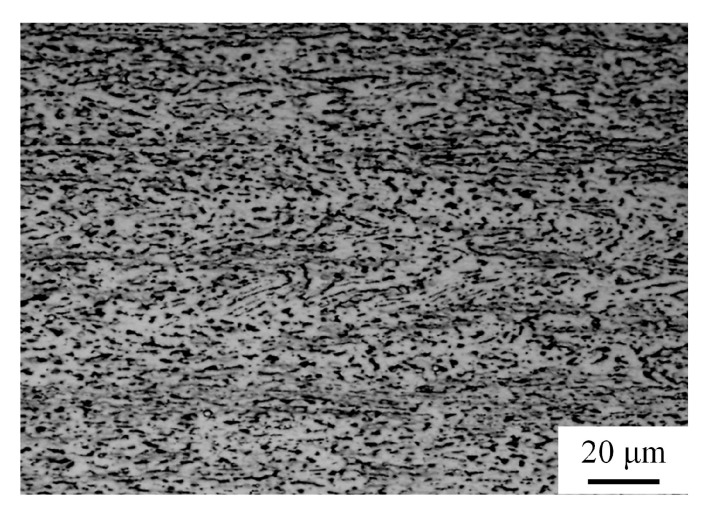
Optical micrograph of as-rolled Ti-Al-4V alloy prior to superplastic tensile deformation.

Figure 2 shows the initial optical microstructure of the as-rolled Ti-6Al-4V alloy prior to tensile deformation, consisting of a mixture of the band-structured β phase (bright contrast) and the dispersed α phase (dark contrast). The mean grain size of the body-centered cubic (bcc) β phase is approximately 6 μm, and the volume fraction of the hexagonal close-packed (hcp) α phase is about 62%.

### 3.2. Tensile Mechanical Behavior of Ti-6Al-4V Alloy

**Figure 3:**
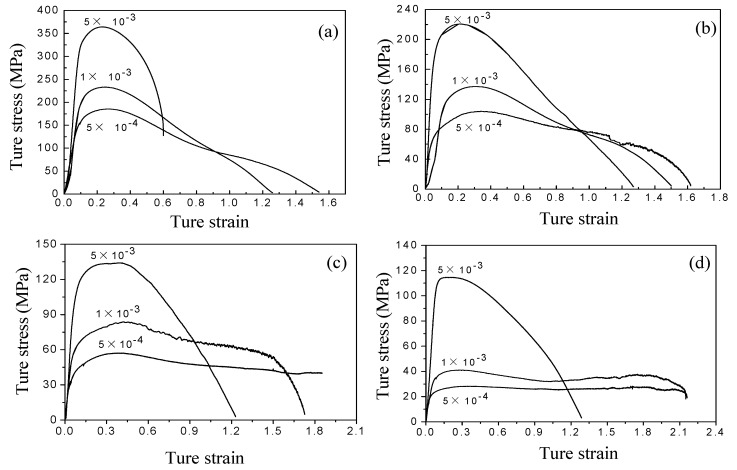
True stress versus strain curves during superplastic tension: (**a**) 973 K; (**b**) 1023 K; (**c**) 1073 K; (**d**) 1123 K.

**Figure 4:**
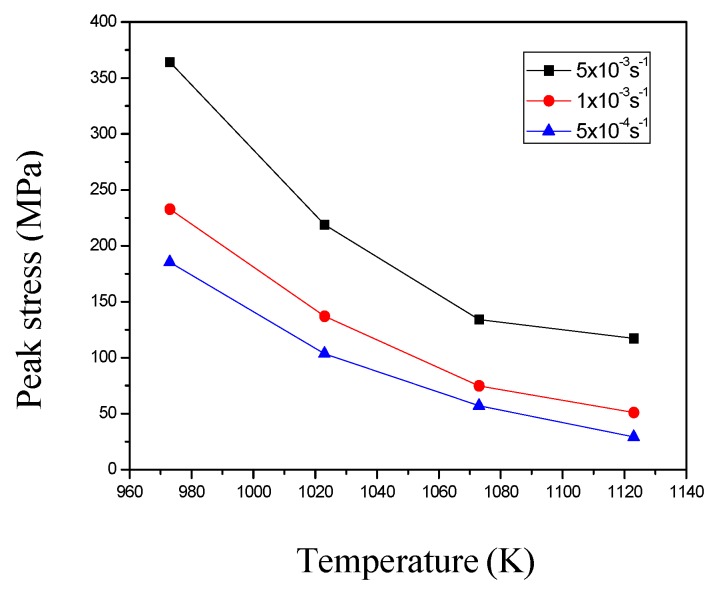
Peak stress as a function of temperature at different initial strain rates.

Figure 3 shows the true stress vs. true strain curves of Ti-6Al-4V alloy at the initial strain rates of 5 × 10^−4^, 1 × 10^−3^, and 5 × 10^−3^ s^−1^ under different deformation temperatures. The changes of peak stress as a function of the deformation temperature and strain rate are shown in Figure 4. The peak stress decreases with decreasing the strain rate at a constant deformation temperature, which reflects the sensitivity of the stress to strain rate. In the case of the same strain rate, the peak stress decreases with increasing deformation temperature. It is worth noting that Ti-6Al-4V alloy deformed at 1123 K with a strain rate of 5 × 10^−4^ exhibits no obvious softening behavior in the strain range of 0.3 to 2.0 (Figure 3d), indicating the typical characteristics of superplastic deformation.

**Figure 5:**
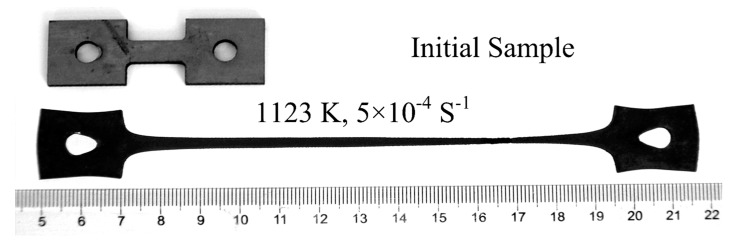
Macrogaraphs of Ti-6Al-4V alloy before and after deformation.

Figure 5 shows the macrograph of the Ti-6Al-4V specimens before and after the tensile tests. The maximum elongation to failure, *δ* = 768%, is obtained in the specimen deformed at 1123 K with an initial strain rate of 5 × 10^−4^ s^−1^. The strain rate sensitivity exponent, *m*, as a function of *δ*, is determined as the following equation [23]:

(1) $$m=\frac{\ln\left(1+\delta \right)}{2+\ln\left(1+\delta \right)}$$

The measured *δ* and calculated m values as a function of deformation temperature and strain rate are shown in Table 1. The m value increases with increasing *δ*, indicating a high strain rate sensitivity resists neck formation and leads to the high tensile elongations in superplastic materials. The m value corresponding to the maximum *δ* value of 768% at 1123 K with a strain rate of 5 × 10^−4^ s^−1^ is about 0.52 greater than 0.5, indicative of a typical superplasticity. The *m* value corresponding to the *δ* value of 82% at 1023 K with a strain rate of 5 × 10^−3^ s^−1^ is 0.23 smaller than 0.3, implying no superplasticity occurs. The m values under other deformation conditions are between 0.39 and 0.52 belonging to the scope of quasi-superplasticity.

### 3.3. Deformation Activation Energy of Ti-6Al-4V Alloy

Superplastic tensile deformation of the Ti-6Al-4V alloy is a thermal activation process. The flow stress as a function of the deformation temperature and strain rate can be expressed by the following formula [24]:

(2) $$\sigma =K\varepsilon^{n}{\dot{\varepsilon }}^{m}\exp\left(\frac{Q}{RT}\right)$$

where *K* is a constant, σ is the flow stress, $\dot{\varepsilon }$ is the strain rate, *n* is the hardening exponent, *m* is the strain rate sensitivity exponent, *R* is the gas constant, *T* is the deformation temperature, and *Q* is the deformation activation energy. When a material is in the superplastic state, *n* ≈ 0. When the strain rate is constant, the following expression about thermal activation energy can be obtained by mathematical manipulation:

(3) $$Q=2.303R\cdot {\left[\partial lg\sigma /\partial \left(1/T\right)\right]}_{\dot{\varepsilon }}{\left[\partial lg\dot{\varepsilon }/\partial lg\sigma \right]}_{T}$$

where ${\left[\partial lg\dot{\varepsilon }/\partial lg\sigma \right]}_{T}=\frac{1}{m}$, thus

(4) $$Q=2.303R\cdot {\left[\partial lg\sigma /\partial \left(1/T\right)\right]}_{\dot{\varepsilon }}\cdot \frac{1}{m}$$

**Figure 6:**
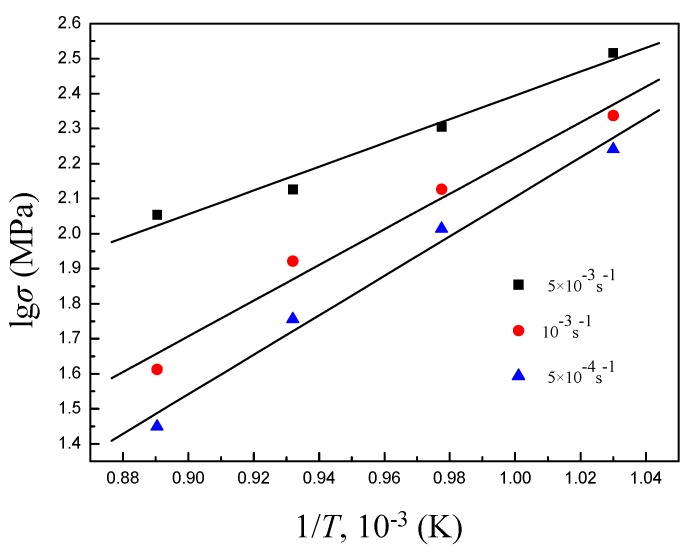
lg*σ* − 1/*T* curve of Ti-6Al-4V alloy.

The data obtained from tensile tests are processed to plot lgσ−1/T curve whose slope is ${\left[\partial lg\sigma /\partial \left(1/T\right)\right]}_{\dot{\varepsilon }}$ which, together with *m* value, is substituted into Equation (4) to get the activation energy, as shown in Figure 6.

Table 2 calculated the activation energies as a function of the deformation temperature and strain rate in Ti-6Al-4V alloy. Superplastic activation energy is close to the grain boundary self-diffusion activation energy in most materials, indicating that superplastic flow is related to grain boundary diffusion. The grain boundary self-diffusion activation energy of Ti-6Al-4V alloy is 130–169 kJ/mol [25]. In the present work, deformation activation energy increases with decreasing the deformation temperature. The activation energy at 973–1073 K is above the grain boundary self-diffusion activation energy, indicating that the grain boundary sliding controlled by the grain boundary diffusion is the dominant mechanism of superplastic deformation. When the deformation temperature is lower than 1073 K, the dislocation movement is much more active at a relatively low deformation temperature, causing a significantly higher activation energy than that at 1123 K. Thus, interfacial sliding during deformation at 973–1073 K is not a dominant mechanism of superplastic deformation; i.e., typical superplastic deformation does not occur, which is consistent with the results reflected by the flow curves in Figure 3. However, a decrease in the deformation temperature leads to the increased flow stress and activation energy, but a high elongation to failure of 536%, which fully displays the potential of superplasticity of Ti-6Al-4V alloy in a low-temperature range.

### 3.4. Microstructures after Tensile Deformation

**Figure 7:**
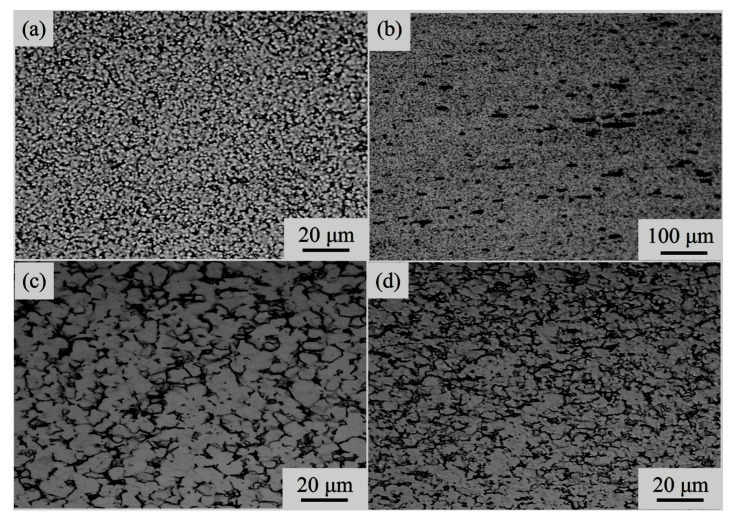
Optical micrographs of Ti-6Al-4V alloy after superplastic tensile deformation at the temperatures of 973 and 1123 K; (**a**) $\dot{\varepsilon }$ = 10^−3^ s^−1^ and (**b**) $\dot{\varepsilon }$ = 5 × 10^−3^ s^−1^ (973 K); (**c**) $\dot{\varepsilon }$ = 10^−3^ s^−1^ and (**d**) $\dot{\varepsilon }$ = 5 × 10^−3^ s^−1^ (1123 K).

Figure 7 show the optical microstructure near the fracture surface of the specimens after the tensile deformation at 973 and 1023 K, respectively. Band-structured β grains before deformation disappear and equiaxed grains are obtained, implying the occurrence of dynamic recrystallization during tensile deformation. Compared with Figure 7c,d, dynamic recrystallization occurs more sufficiently and grains become more equiaxed at a low strain rate. According to Figure 7a,c, the grain size of the β phase significantly increases with an increasing deformation temperature under the same strain rate, mainly due to the easier growth of recrystallized β grains at a relatively higher temperature.

When the deformation temperature is low and the strain rate is high, many cavities near the fracture surface appear. These cavities are small in volume, many in quantity, and distributed along the tensile direction, as shown in Figure 7b. The existence of these cavities results in the failure of the material. The mean grain size of a body-centered cubic (bcc) β phase is approximately between 2.3–21 μm (973 K) and 10.3–37.2 μm (1123 K), the mean grain size of face-centered cubic (fcc) α phase is approximately between 2–18 μm (973 K) and 3.6–13.02 μm (1123 K).

### 3.5. Theoretical Forecast of Low-Temperature Deformation Mechanism Maps Incorporating Dislocation Quantity

Deformation mechanism maps are used extensively in the field of high temperature creep to provide simple visual displays of the dominant creep mechanisms over a selected range of experimental conditions. The concept of mapping the flow processes may be traced to an early proposal for the construction of a creep diagram and the subsequent quantification of this proposal in the form of deformation mechanism maps plotting the normalized stress, *σ*/*G*, against the homologous temperature, *T*/*T_m_*. For the present experiments, it is more convenient to use alternative forms of deformation mechanism maps in which the normalized grain size, *d*/*b*, is plotted against the normalized stress, *σ*/*G*, at a constant temperature or the normalized stress, *σ*/*G*, is plotted against the reciprocal of the homologous temperature, *T_m_*/*T*, at a constant grain size. Very little information is available at present on the construction of deformation mechanism maps for incorporating the dislocation quantity inside the grain metals processed using Low-temperature superplasticity.

In the present work, a new deformation mechanism map containing the dislocation density has been proposed for the Ti-6Al-4V alloy to predict its low-temperature deformation mechanism, and to analyze quantitatively the dislocation quantity during its deformation.

#### 3.5.1. Construction of a Deformation Mechanism Map Incorporating the Dislocation Quantity

Taking the Burgers vector compensated grain size as the Y-axis and taking the modulus compensated flow stress as the X-axis, authors construct the deformation mechanism maps at different temperature controlled strain rates. Each deformation mechanism can be expressed by a rate controlling equation as follows [17,26]:

(5) $$\gamma_{ij}=f_{ij}\left(\sigma ,T,S_{i},P_{j}\right)$$

where *S_i_* is the state variable that describes the current microstructural state, and *P_j_* is the material properties, such as lattice parameter, atomic volume, binding energy, modulus, and rate controlling diffusion coefficient, etc. According to the different deformation mechanisms, *S_i_*, *P_j_* can be different values and algebraic expressions.

Specifically, a general deformation mechanism can be expressed by the following constitutive equation [17,26]:

(6) $$\dot{\varepsilon }=A_{i}{\left(\frac{b}{d_{i}}\right)}^{p}\cdot \frac{D}{K\cdot T\cdot b^{2}}\cdot {\left(\frac{\sigma_{i}}{E}\right)}^{n}$$

where $\dot{\varepsilon }$ is the steady strain rate; $A_{i}$, *n*, *P* are material constants, depending on the deformation mechanism; $\sigma_{i}$ is the true stress; *E* is the Young’s modulus; $d_{i}$ is the grain size; *b* is the Burgers vector; *D* is the diffusion coefficient that equals to the lattice diffusion coefficient *D_L_*, or the dislocation pipe diffusion coefficient *D_P_*, or the grain boundary diffusion coefficient *D_gb_*.

Data corresponding to different deformation mechanisms are substituted into Equation (6), and three kinds of constitutive equations including diffusion flow mechanism, grain boundary sliding, and slip can be obtained. These equations were used to calculate the boundaries and nodal points of the different mechanism regimes. Computer software was also applied to plot the RWS deformation mechanism map.

The calculation model of the dislocation quantity in a single grain is as follows [27]:

(7) $$n_{i}=2\left[\left(1-\nu \right)\cdot \pi \cdot d_{i}\cdot \tau_{i}\right]/\left(Gb\right)$$

where $n_{i}$ is the dislocation quantity inside the grain, *ν* is Poisson’s ratio, $\tau_{i}$ is the shear stress, $\tau_{i}=0.5\sigma_{i}$. Tensile data of Ti-6Al-4V alloy at low temperatures are substituted into Equation (7) to solve the dislocation quantities at various nodal points by integrating the RWS (Ruano–Wadsworth–Sherby) deformation mechanism map. Thus, new deformation mechanism maps of Ti-6Al-4V alloy containing the dislocation quantity can be obtained. The basic physical parameters of the titanium alloy used for the calculation are as follows (Table 3):

#### 3.5.2. Analysis of Low-Temperature Tensile Behavior Using Deformation Mechanism Maps Containing Dislocation Quantities

**Figure 8:**
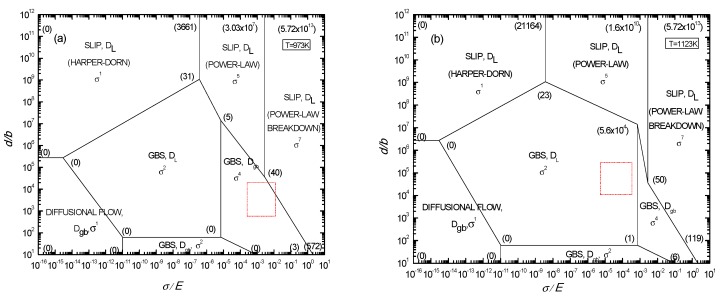
Rate controlling deformation mechanism maps for two-phase titanium alloy constructed at 973 K (**a**) and 1123 K (**b**).

The deformation mechanism maps of two-phase titanium alloy at 973 and 1123 K were plotted according to the above method, as shown in Figure 8a,b, respectively. The Burgers vector compensated grain size, modulus compensated stress, and dislocation quantity of Ti-6Al-4V alloy during tensile deformation at 973 and 1123 K, respectively, were calculated. Deformation mechanisms of Ti-6Al-4V alloy are determined based on the calculated results from Figure 8a,b. As shown in Figure 8, the data in parenthesis of a nodal point is the dislocation quantity. The mechanism regime is surrounded by one polygon with dislocation quantity nodal points. If experimental data, such as normalized grain size and stress, and calculated dislocation quantity fall into a certain regime, the corresponding dominant mechanism in this regime can be forecasted. After data falls into dislocation polygon, name, diffusion coefficient, and stress exponent, which are used to characterize the rate controlling the deformation mechanism.

According to the flow curves at deformation temperatures of 973 and 1123 K in Figure 3, the scope of normalized flow stress $(\sigma /E)\times 10^{4}$ is determined to be between 3.2–55.1 and 2.8–21.7. The scope of the Burgers vector compensated grain size $(d/b)\times 10^{-4}$ was calculated to be between 0.8–7.2 and 3.6–13.02, based on the grain size in micrographs (Table 4). The box areas in Figure 8a,b are the corresponding deformation mechanism regimes at 973 and 1123 K for different strain rates.

**Figure 9:**
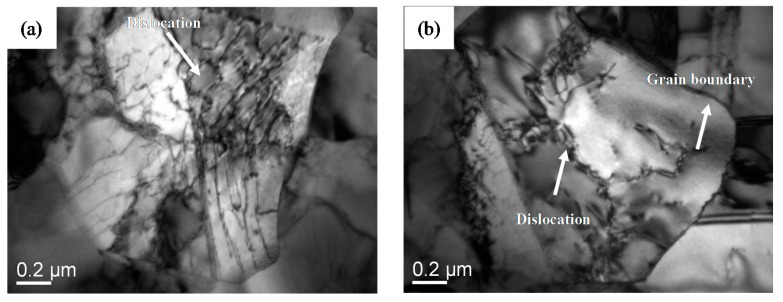
Transmission electron microscopy (TEM) microstructures of superplastic deformation for Ti-6Al-4V alloy at 973 K (**a**) and 1123 K (**b**).

It can be seen from Figure 8a that the data at 973 K with low strain rates falls into the dislocation polygon (0) (0) (3) (40) (5), indicating that the stress exponent is 4, and the corresponding deformation mechanism is the dislocation pipe grain boundary sliding. With increasing strain rate, the data fall into the dislocation polygon (40) (3) (572) (5.72 × 10^13^) (3.03 × 10^7^), indicating that the stress exponent is 7, and the corresponding deformation mechanism is the dislocation slip. Therefore, the deformation mechanism at 973 K gradually changes from the dislocation pipe grain boundary sliding to the dislocation slip with increasing strain rate. Figure 9a show the transmission electron microscopy (TEM) microstructures near the fracture surface of the specimens after tensile deformation at 973 K.

It can be also seen from Figure 8b that the data at 1123 K for different strain rates fall into dislocation polygon (0) (0) (1) (5.6 × 10^4^) (23), indicating that the stress exponent is 2, and the corresponding deformation mechanism is a grain boundary sliding controlled by lattice diffusion.

TEM micrographs of superplastic deformation for Ti-6Al-4V alloy at 973 and 1123 K with an initial strain rate of 5 × 10^−4^ are shown in Figure 9. Figure 9a shows that large amounts of dislocations exist in α phase grains, which indicates that the dislocation movement plays an important role in superplastic deformation of as-received alloy. Compared with Figure 8a,b and Figure 9b, by increasing the deformation temperature the regime controlled by the superplastic grain boundary sliding significantly expands, while the dislocation pipe grain boundary sliding decreases. Then lots of dislocations are seriously consumed, a large grain boundary is formed, which is conducive to the grain boundary slip. This indicates that superplastic grain boundary sliding is the dominant deformation mechanism at a relatively high deformation temperature. These results are in agreement with those from the calculation and analysis of the activation energy.

Ti-6Al-4V titanium alloy consists of α and β phases in which the proportion of β phase is 38%. α phase is a hcp crystal structure with three slip systems in single cell; β phase is a bcc crystal structure with twelve slip systems in single cell. During superplastic deformation, the slip systems in β phase firstly operate; β phase can accommodate effectively the stress concentration, eliminate partial deformation resistance, and hence restrain the propagation of the crack source at a stress concentration during the deformation. In the meantime, the coexistence of a dual phase will restrain the deformation-induced grain growth. It is noted in Figure 2 and Figure 7 that no obvious grain growth occurs in the present alloy prior to and following superplastic deformation, and the grain shape tends to be equiaxed. Fine and equiaxed grains help the grain boundary from sliding during deformation Therefore, under the circumstance of not withstanding fine grain processing and at a temperature lower than the conventional superplastic temperature of the present alloy, the Ti-6Al-4V alloy exhibits good superplasticity.

## 4. Conclusions

The results demonstrate that Ti-6Al-4V alloy shows excellent superplasticity in the temperature ranging from 973 to 1123 K over a strain rate 5 × 10^−4^–10^−3^ s^−1^, where the strain rate sensitivity exponent (*m*) varies from 0.23 to 0.52 and the deformation activation energy (*Q*) falls in the range of 106.70–402.61 kJ/mol. A maximum elongation to failure rate of 768% has been achieved with *m* reaching its peak value at 0.52 and *Q* nearing the grain boundary self-diffusion activation energy. *Q* decreases gradually when raising the deformation temperature and it becomes higher than the grain boundary self-diffusion activation energy when the temperature is between 973 and 1023 K. This shows that Ti-6Al-4V alloy has good plastic forming capability. In the deformation mechanism maps incorporating the dislocations inside the grains created, they illustrate the quantitative relationships among flow stress, grain size, and dislocation quantity, and accurately predict the superplastic deformation mechanism of Ti-6Al-4V alloy in a low-temperature range. The low-temperature superplastic tensile deformation mechanisms of Ti-6Al-4V alloy are a grain boundary slip induced by dislocation movement. At different strain rates, the dislocation sources in different directions of the Burgers vector in the grain start to form the grain boundary slip under different stress indices.

## Figures and Tables

**Table 1:** Measured *δ* and calculated *m* values as a function of deformation temperature and strain rate in Ti-6Al-4V alloy.

Temperature *T*, (K)	Initial Strain Rate $\dot{\varepsilon }$, (s^−1^)	Strain Rate Sensitivity Exponent *m*	Elongation to Failure *δ*, (%)
1123	5 × 10^−3^	0.39	263
1123	10^−3^	0.52	758
1123	5 × 10^−4^	0.52	768
1073	5 × 10^−3^	0.38	240
1073	10^−3^	0.46	466
1073	5 × 10^−4^	0.48	536
1023	5 × 10^−3^	0.39	256
1023	10^−3^	0.43	347
1023	5 × 10^−4^	0.45	406
973	5 × 10^−3^	0.23	82
973	10^−3^	0.39	252
973	5 × 10^−4^	0.43	366
973	3 × 10^−4^	0.43	359

**Table 2:** Calculated activation energies as a function of deformation temperature and strain rate in Ti-6Al-4V alloy.

Initial Strain Rate $\dot{\varepsilon }$, (s^−1^)	Temperature *T*, (K)			
	973	1023	1073	1123
5 × 10^−3^	242.46	228.80	184.60	106.70
10^−3^	363.72	343.23	276.92	160.06
5 × 10^−4^	402.61	379.93	306.53	177.18
Average	336.26	317.32	256.02	147.98

**Table 3:** Physical parameters of Ti-6Al-4V alloy [28].

*b_α_* = 2.5 × 10^−10^ m	$G_{\alpha }=4.36\times 10^{4}\left[1-1.2\frac{\left(T-300\right)}{1933}\right]$	
*b_β_* = 2.86 × 10^−10^ m	$G_{\beta }=2.05\times 10^{4}\left[1-0.5\frac{\left(T-300\right)}{1933}\right]$	
ν=0.34	*K* = 1.38 × 10^−23^ J/K	*E* = 2*G*(1 + *ν*)

**Table 4:** Calculated results for the superplastic deformation of Ti-6Al-4V alloy.

*T* (K)	(*d*/*b*) × 10^−4^	(*σ*/*E*) × 10^4^	*έ* (10^−4^·s^−1^)
973	0.8–7.2	3.2–55.1	5–50
1123	3.6–13.02	2.8–21.7	5–50
